# Microplastics and Nanoplastics in Cardiovascular Disease: An Emerging Cardiovascular Risk Factor

**DOI:** 10.1007/s12012-026-10112-z

**Published:** 2026-04-13

**Authors:** Alberto Polimeni, Domenico Simone Castiello, Rossella Quarta, Tommaso Gori, Giovanni Martino, Letizia Rosa Romano, Federica Meringolo, Giovanni Lopes, Masakazu Yasuda, Ciro Indolfi, Antonio Curcio

**Affiliations:** 1 Division of Cardiology, Department of Pharmacy, Health and Nutritional Sciences, University of Calabria, Rende, Italy; 2 Division of Interventional Cardiology, Annunziata Hospital, Cosenza, Italy; 3 Division of Cardiology, Annunziata Hospital, Cosenza, Italy; 4 Department of Advanced Biomedical Sciences, University of Naples Federico II, Naples, Italy; 5 Department of Cardiology, Cardiology I, University Medical Center, Johannes Gutenberg-University Mainz, Mainz, Germany; 6 Division of Cardiology, Department of Medical and Surgical Sciences, Magna Graecia University, Catanzaro, Italy; 7 Division of Cardiology, Sakurabashi Watanabe Advanced Healthcare Hospital, Osaka, Japan

**Keywords:** Microplastics, Nanoplastics, Cardiovascular risk, Inflammation, Atherosclerosis, Cardiac remodeling

## Abstract

Microplastics and nanoplastics (MNPs) have become pervasive contaminants in air, water, and food, leading to continuous human exposure. Contrary to the long-standing assumption that these particles are biologically inert, accumulating evidence now shows that MNPs cross epithelial and endothelial barriers, circulate systemically, and accumulate in cardiovascular tissues. Experimental, translational, and early clinical studies demonstrate that some MNPs can induce oxidative stress, endothelial dysfunction, mitochondrial injury, immune activation, and coagulation pathway dysregulation, mechanisms that converge on atherosclerotic development and progression, plaque destabilization, atherothrombosis, and cardiac remodeling. Recent human data identifying MNPs in carotid and coronary plaques, arterial specimens, thrombi, and cardiac tissues provide direct proof-of-concept for their cardiovascular involvement. These findings position MNPs as an emerging cardiovascular risk factor, with implications for prevention, patient risk stratification, and environmental health policy. Major knowledge gaps remain, including the absence of standardized biomarkers of exposure, dose–response quantification, and longitudinal clinical cohorts. Recognizing environmental plastics as cardiovascular toxins demands urgent, multidisciplinary investigation and integration of environmental exposure into cardiovascular disease prevention frameworks.

**Graphical Abstract:**

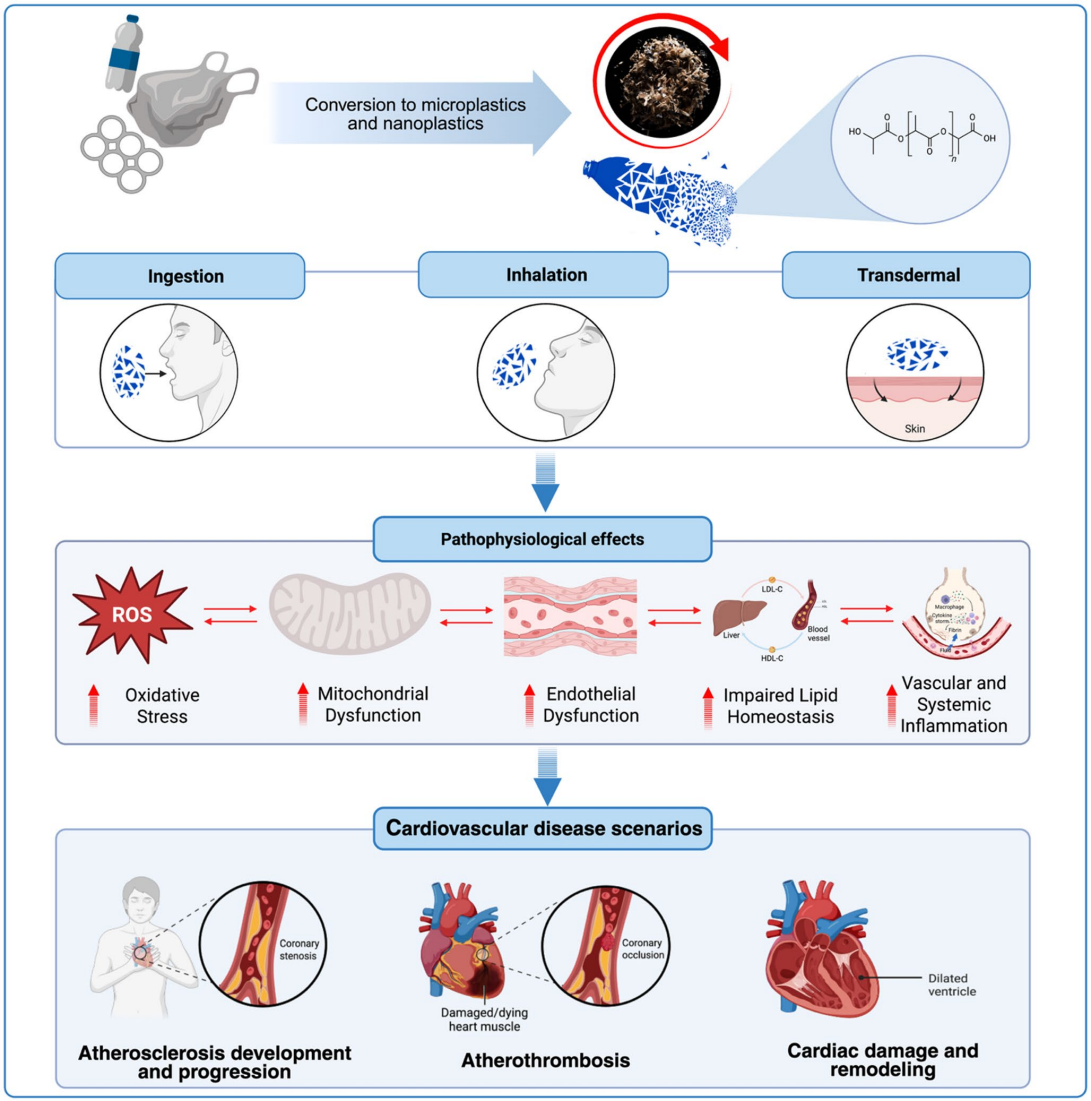

Overview of exposure routes and subsequent pathophysiological mechanisms of microplastics and nanoplastics leading to cardiovascular disease scenarios.

## Introduction

Microplastics and nanoplastics (MNPs) have rapidly emerged as pervasive environmental contaminants with direct relevance to human physiology. Once regarded as biologically inert, it is now clear that these particles can enter the bloodstream, cross cellular barriers, accumulate in tissues, and trigger cellular stress responses. Their effects have been well documented in marine and ecological systems, but only recently have mechanistic and clinical data revealed their implications for the cardiovascular system [1].

Cardiovascular injury induced by MNPs converges on four core pathways: oxidative stress, endothelial dysfunction, chronic inflammation, and mitochondrial dysregulation [2–4].

These processes are recognized drivers of atherosclerotic plaque initiation and progression, plaque instability and thrombosis, and myocardial structural and functional remodeling.

Importantly, MNPs have now been detected in human arteries, atherosclerotic plaques, coronary thrombi, saphenous vein grafts, pericardium, and myocardium, providing direct evidence of their bioavailability within cardiovascular tissues [1, 5, 6]. These human findings, together with supportive experimental evidence, suggest that MNPs represent an emerging cardiovascular risk factor, bridging environmental science and cardiovascular medicine.

This review synthesizes current mechanistic, translational, and early clinical evidence linking MNP exposure to cardiovascular disease. Furthermore, we aim to integrate experimental and emerging human data within a structured translational framework, critically examining both biological plausibility and clinical implications.

In order to support the concept of an association between MNPs and cardiovascular disease, we approach this narrative review using a revised adaptation of Koch’s four classical postulates, originally formulated in the late nineteenth century to establish causality between a microorganism and an infectious disease. In their original form, Koch’s postulates state that: (1) the suspected pathogen must be consistently present in individuals with the disease; (2) it must be isolated and characterized; (3) experimental introduction of the agent into a susceptible host should reproduce the disease; and (4) the same agent must then be re-isolated from the experimentally affected host. Although these criteria were developed for infectious diseases, they provide a conceptual framework for evaluating causality [7]. Here, we apply an adapted version of this logic to environmental exposure, examining whether MNPs can be identified, detected in affected tissues, mechanistically linked to cardiovascular injury, and associated with clinical disease. By organizing the available evidence according to adapted causality criteria and exploring potential interactions with established cardiovascular therapies, we aim to provide a clinically oriented synthesis that may help guide future research and risk stratification strategies. Finally, we discuss the need for future studies to further elucidate the possibility that MNPs may be a novel definite risk factor for cardiovascular diseases.

## Can the Causal Agent Be Identified?

### Epidemiology of Plastics

Global plastic production has increased exponentially, exceeding 400 million tons per year and is projected to double by 2050. This sustained growth reflects plastic’s low cost, versatility, and widespread use across consumer products, industrial applications, healthcare, and food packaging [1]. However, the same characteristics that make plastic advantageous also underlie its environmental persistence: plastics are not biodegradable and undergo slow fragmentation into MNPs once released into the environment [8].

Plastic waste accumulation has resulted in ubiquitous environmental contamination. MNPs are now detected in oceans, rivers, soil, drinking water, indoor and outdoor air, and food supplies. For example, resuspension from sea spray and aerosolization from urban dust and textiles contribute to continuous atmospheric circulation of MNPs [9, 10]. Human exposure is therefore chronic and unavoidable.

Bottled water, processed salt, seafood, and ultra-processed food packaging represent major dietary sources of MNP exposure. In particular, marine organisms can bioaccumulate microplastics (MPs).

through trophic transfer within contaminated aquatic ecosystems. Filter feeders such as mussels and oysters, as well as fish species consumed whole or with retained gastrointestinal content, may contain detectable MP burdens [11, 12]. Thus, food itself, beyond packaging-related contamination, constitutes a relevant pathway of oral exposure. At the same time, household environments remain the dominant source of inhalation exposure [9].

This epidemiological context challenges the historical assumption that plastic exposure is biologically harmless. Instead, the scale and continuity of exposure indicate that MNPs represent a persistent, population-wide environmental burden, positioning them as a potential cardiovascular risk factor of global relevance [13].

### Physicochemical Characteristics of Microplastics and Nanoplastics

**Fig. 1:**
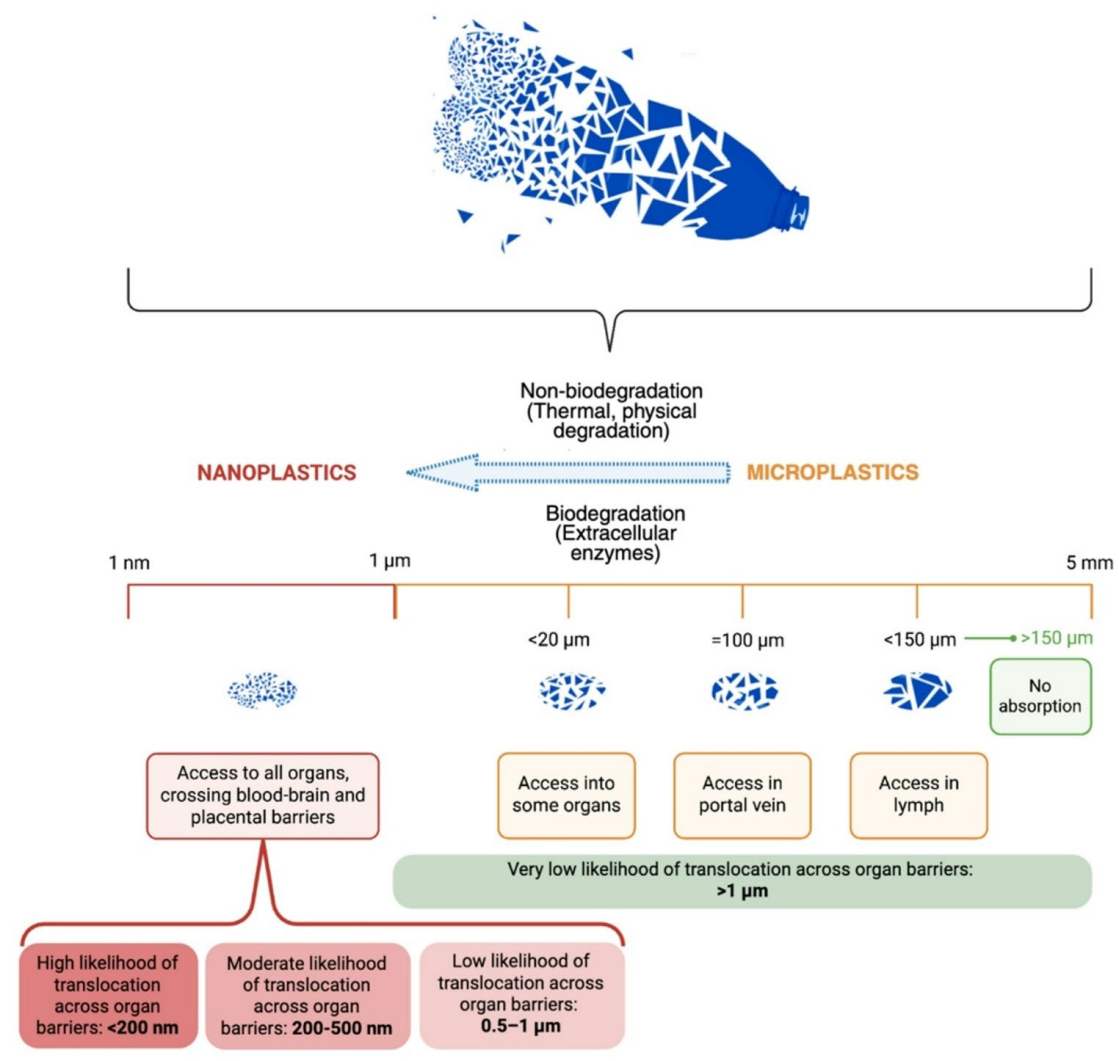
Classification of microplastics and nanoplastics with different capacity of absorption

MPs are plastic particles measuring less than 5 mm, whereas nanoplastics (NPs) are less than 1 μm in size (Fig. 1). They form a heterogeneous class of environmental contaminants, differing in size, shape, polymer composition, surface charge, and degree of weathering, factors that strongly influence biological behavior and toxicity [14].

MNPs originate as primary particles, intentionally manufactured for use in cosmetics, pharmaceuticals, industrial abrasives, and medical products, or as secondary particles, generated through the mechanical abrasion and chemical degradation of larger plastic waste [15, 16]. Polyethylene (PE), polypropylene (PP), and polyethylene terephthalate (PET) are among the most frequently detected polymers in environmental and human biological samples, reflecting their high global production volume and widespread consumer use. Polystyrene (PS), although less consistently reported as a dominant polymer in biomonitoring studies, remains the most commonly used material in experimental in vitro and in vivo models due to its physicochemical stability, commercial availability in standardized particle sizes, and reproducibility. This discrepancy between experimental predominance and real-world exposure patterns should be considered when interpreting mechanistic findings derived primarily from PS-based systems [17–19]. Finally, polyvinyl chloride (PVC), despite its substantial global production, is less consistently reported in human biomonitoring studies [20].

NPs are particularly concerning due to their large surface area-to-volume ratio, greater surface reactivity, and enhanced ability to adsorb metals, endocrine disruptors, and persistent organic pollutants. These chemical cargoes may amplify inflammatory, oxidative, and endocrine-disrupting effects beyond the impact of the polymer itself [21].

Size and surface chemistry critically determine how MNPs interact with biological systems. Smaller particles more readily cross epithelial barriers, undergo cellular internalization, and reach the circulation. Positively charged particles show higher tissue uptake due to electrostatic interactions with negatively charged cellular membranes [22].

Experimental studies demonstrate that exposure to MNPs can elevate reactive oxygen species production, disrupt antioxidant defense systems, interfere with lipid metabolism, and alter gene expression programs linked to cellular stress and senescence. The degree of toxicity is influenced by particle dose, duration of exposure, polymer type, and co-transported chemical contaminants [23].

Taken together, the physicochemical diversity and biological reactivity of MNPs suggest that they should not be viewed as inert materials, but rather as biologically active agents capable of influencing cardiovascular physiology and pathology [3, 24]. Although MNPs have been identified in multiple organ systems, including pulmonary, gastrointestinal, and neurological tissues [25], the cardiovascular system is uniquely positioned at the intersection between environmental exposure and systemic disease, given its central role in inflammatory signaling, endothelial integrity, and organ perfusion [26]. Given the global burden of cardiovascular disease [27], understanding potential environmental contributors is of particular importance.

## Can plastics be Identified in Tissues? Exposure Routes and Human Biodistribution

### Ingestion, Inhalation, Dermal Contact

**Fig. 2:**
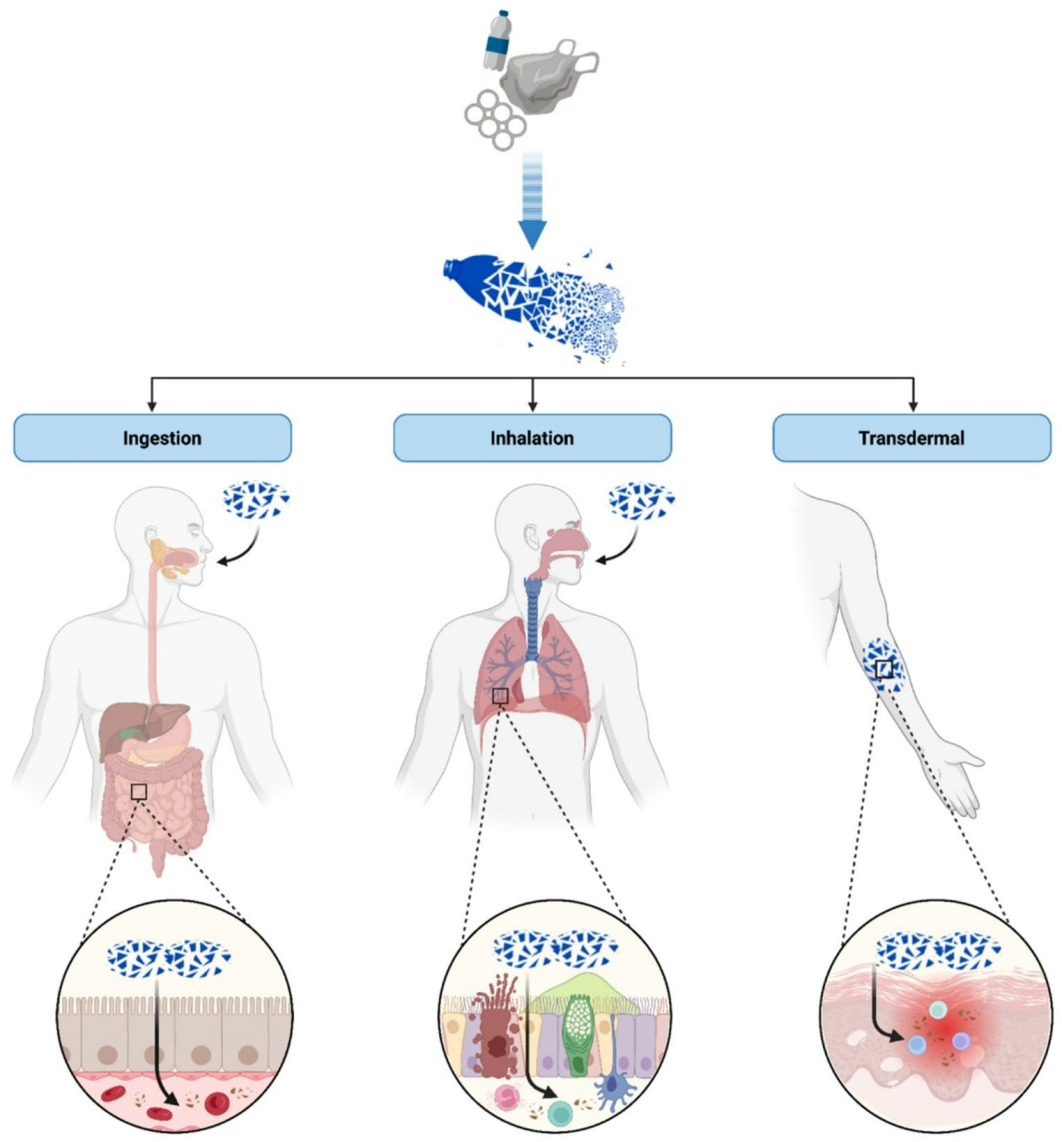
Exposure routes of microplastics and nanoplastics. Inhalation, ingestion and transdermal absorption are described as entrance door of microplastics and nanoplastics entrance in the blood circulation

Humans are continuously exposed to MNPs primarily through ingestion, inhalation, and, to a lesser extent, dermal contact (Fig. 2). Recent estimates suggest that an adult may inhale 0.21–2.51 × 10⁶ particles per year, drinks 0.22–1.20 × 10⁶ particles per year and ingest an additional 0.49–1.20 × 10⁶ particles through food and water, with indoor air exposure typically exceeding outdoor exposure due to synthetic textiles and particulate resuspension [28].

The extent of internalization and tissue distribution depends on particle size, surface charge, hydrophobicity, and interaction with biological membranes.

Ingestion represents a major exposure pathway, driven by contamination of drinking water, bottled beverages, sea salt, seafood, and processed foods. Within the gastrointestinal tract, experimental studies indicate that sub-micron and nanoscale particles may cross the intestinal epithelium via transcytosis, M-cell transport, paracellular leakage, and uptake by intestinal dendritic cells. In murine models, continuous oral exposure to PS MNPs has been shown to induce gut microbiota dysbiosis and impairment of intestinal barrier function, facilitating systemic translocation of particles beyond the gastrointestinal tract [29, 30]. Conditions associated with increased intestinal permeability may theoretically amplify this process, although direct human evidence remains limited.

Inhalation contributes substantially to internal exposure, particularly in indoor environments, where concentrations of airborne fibers and fragments are higher than outdoors due to synthetic textiles, upholstered materials, and dust resuspension. Even if particles < 2.5 μm (PM2.5) can deposit in alveolar regions, systemic translocation across the alveolar–capillary barrier appears to be largely restricted to the ultrafine fraction (generally < 500 nm) [31]. These nanoscale particles via endocytosis by epithelial cells, phagocyte-mediated transport, or paracellular diffusion under conditions of barrier disruption [32]. In addition to direct alveolar translocation, particles deposited in the upper and conducting airways may undergo mucociliary clearance toward the oropharynx, followed by swallowing [33]. This mucociliary escalator mechanism represents a secondary pathway linking inhalation to gastrointestinal exposure, particularly for larger particles (> 2.5 μm) that are less likely to reach the alveolar compartment [15]. Thus, inhalation may contribute indirectly to intestinal MNP burden in addition to direct respiratory effects. Similar translocation phenomena have been extensively documented in the fields of air pollution and nanotoxicology. Ultrafine particles (UFPs) and engineered nanoparticles have been shown to cross pulmonary and gastrointestinal barriers, enter systemic circulation, and accumulate in secondary organs including the heart, liver, and brain [34, 35]. Experimental and epidemiological studies have linked exposure to nanoscale particulate matter (PM2.5 and UFPs) with endothelial dysfunction, systemic inflammation, oxidative stress, and increased cardiovascular morbidity and mortality [36, 37]. These data provide a broader mechanistic framework supporting the biological plausibility of systemic effects induced by inhaled or ingested nano-sized particles, including MNPs.

Dermal uptake is generally limited, but NPs < 50 nm may penetrate inflamed or barrier*-*disrupted skin, and lipophilic surface coatings can enhance transdermal permeability [38].

Internalized particles accumulate in endosomes, lysosomes, mitochondria, and the cytosol. Failure of lysosomal degradation leads to lysosomal membrane permeabilization, mitochondrial stress, and reactive oxygen species (ROS) generation [39].

These mechanistic pathways support the growing body of evidence that MNPs can enter the bloodstream and, in some contexts, accumulate within cardiovascular tissues. Human studies have identified MNPs within atherosclerotic plaques and thrombotic material, suggesting that translocated particles may not only circulate but also localize within diseased vascular compartments, enabling direct interaction with endothelial, immune, and myocardial cells [40–42].

### Accumulation and Impact in Cardiovascular Tissues

Once MNPs enter the circulation, their distribution and persistence in tissues appear to be influenced by particle size, charge, surface properties, and protein corona composition. Experimental models indicate that smaller and positively charged particles show higher rates of vascular uptake and longer residence times within tissues [43, 44].

In vitro studies demonstrate that endothelial cells, vascular smooth muscle cells, macrophages, and circulating immune cells can internalize MNPs through mechanisms such as clathrin-mediated endocytosis, caveolar transport, and phagocytosis. Cellular uptake has been associated with oxidative stress, mitochondrial dysfunction, and activation of inflammatory signaling pathways [18].

In vivo animal studies have shown that ingested or inhaled MNPs may reach the vascular wall, myocardium, and perivascular tissues. Rodent models report myocardial fiber disarray, interstitial fibrosis, endothelial swelling, capillary congestion, and increases in inflammatory cell infiltration [45]. These changes appear to involve NLR family pyrin domain containing 3 (NLRP3) inflammasome activation, endothelial nitric oxide synthase (eNOS) uncoupling, and mitochondrial oxidative stress [46, 47].

Vascular exposure enhances platelet reactivity, promotes thrombosis, stiffens large arteries and thickens the arterial wall [48, 49], illustrated by pigment-laden MPs in human thrombi [6].

From a human perspective, recent analytical advances have enabled the identification of MNPs in arterial tissue, cardiac specimens, thrombotic material, pericardial samples, and saphenous vein grafts [50–52]. Carotid plaques show a higher polymer burdens than unaffected aorta, along with elevated inflammatory mediators and reduced collagen, and the presence of MNP-positive plaques predicts three-year incidence of myocardial infarction, stroke and all-cause mortality [5]. More recently, human femoral artery atherosclerotic plaques were shown to contain significantly higher levels of MNPs compared with non-diseased arterial tissue, further supporting the presence of synthetic polymers across different vascular territories [42]. Although these findings do not establish causality, they provide direct evidence that MNPs are present within cardiovascular tissues.

Taken together, the current experimental and early clinical observations suggest that MNPs may accumulate preferentially at sites of vascular injury or inflammation, potentially interacting with cellular pathways relevant to atherosclerotic disease progression. However, an important unresolved question concerns the directionality of this association. While MNPs have been detected within atherosclerotic plaques [5, 42], current human data are largely cross-sectional and cannot determine whether MNP accumulation precedes plaque formation or whether established plaques create a microenvironment that facilitates secondary particle retention. Atherosclerotic lesions are characterized by lipid-rich necrotic cores, extracellular matrix remodeling, and altered permeability, all of which could theoretically favour passive entrapment of circulating particles [53]. Thus, a “chicken-and-egg” dilemma persists. Additional research is needed to clarify causal inference, dose–response relationships, temporal dynamics of tissue deposition, and whether tissue accumulation contributes directly to clinical cardiovascular outcomes.

### Toxicokinetics and Dose-Response Relationships

Pre-clinical data show a clear exposure–response gradient. In mice, 42-day oral administration of PS-NPs at concentration of 30, 60 and 100 mg L⁻¹ in drinking water produced dose-dependent declines in left-ventricular ejection fraction, ventricular dilation, wall thinning, bradycardia and Tumor Necrosis Factor- α (TNF-α)/Nuclear Factor kappa-light-chain-enhancer of activated B cells (NF-κB) pathway activation [54]. However, based on estimated daily water intake in mice (approximately 4–7 mL/day for a 25 g animal), the highest concentration tested (100 mg/L) corresponds to an approximate exposure of ~ 15–25 mg/kg/day. Using standard body surface area scaling, this would translate to a human equivalent dose of roughly 1–2 mg/kg/day, substantially exceeding current estimates of environmental human intake. These experimental paradigms may therefore reflect high-dose mechanistic models rather than typical chronic environmental exposure levels. Nevertheless, such high-dose models may help delineate biological pathways that could also be relevant under chronic low-dose conditions.

In a murine inhalation model, airborne PS-NPs administered via controlled aerosol exposure accumulated in myocardial tissue and were associated with extracellular matrix remodeling and activation of phosphatidylinositol 3-kinase/protein kinase B (PI3K/AKT) signaling pathways [55].

In ApoE⁻/⁻ mice orally exposed to PS for several weeks, vascular smooth muscle cell migration was enhanced via kinesin family member 15 (KIF15) upregulation, accelerating atherosclerotic plaque progression [56]. In vitro exposure of neonatal rat cardiomyocytes to PS-NPs displayed concentration-dependent contractile loss and mitochondrial depolarisation [57]. Additional models reveal dose-dependent cardiac accumulation culminating in fibrosis, bradyarrhythmia and broader cardiovascular dysfunction [17, 58]. MNP surface chemistry modulates haemostatic toxicity: in a murine model, intravenous exposure to aminated PS accelerates thrombogenesis and shortens clotting time, whereas carboxylated counterparts form smaller clots [17, 59].

Beyond dose-response relationships, toxicokinetic parameters critically influence biological impact. Experimental murine models suggest that absorption efficiency is strongly size-dependent, with nanoscale particles demonstrating greater translocation across epithelial barriers compared with larger MPs [60]. Following systemic entry, particles distribute preferentially to highly perfused organs, including liver, spleen, and, under certain conditions, vascular and cardiac tissues. Limited data indicate that smaller particles may exhibit prolonged tissue retention, whereas larger particles are more likely to be sequestered by the mononuclear phagocyte system [61]. However, quantitative human dose-response data on human absorption rates, bioaccumulation kinetics, metabolic transformation, and clearance pathways remain sparse. The extent to which chronic low-dose exposure leads to cumulative cardiovascular burden has yet to be defined, highlighting the need for longitudinal biomonitoring and reinforcing that sustainable plastics management has become a global priority.

## Is There a Biological Rationale? Molecular Mechanisms of Cardiovascular Toxicity

The MNPs exposure has been demonstrated and associated with several mechanisms of cardiovascular toxicity in both animal and human studies (summarized in Tables 1 and 2, respectively). From a pathophysiological view, oxidative stress, endothelial dysfunction, mitochondrial dysregulation and inflammation are well recognized processes associated with cardiovascular toxicity [18]. However, it is important to acknowledge that current evidence linking MNP exposure to these mechanisms derives predominantly from experimental models, and the precise hierarchy and integration of these pathways in human disease remain incompletely defined. Furthermore, and perhaps most importantly, it should be underscored that the experimental literature on MNP-induced cardiovascular effects is highly heterogeneous. Studies differ substantially with respect to polymer type (most commonly PS-based models), particle size (ranging from tens of nanometres to several micrometres), exposure route (oral, inhalational, intravenous, or direct in vitro incubation), administered dose, and biological model (murine, rat, zebrafish, or isolated human cells). These variables critically influence cellular uptake, biodistribution, and biological response. Consequently, mechanistic findings should be interpreted within the specific experimental context in which they were generated, and caution is warranted when extrapolating across models or to human disease.

**Table 1:** Principal animal studies about MNPs detection in cardiovascular tissues with the associated molecular mechanism and the potential pathophysiological and clinical effect

Study: first author, year	Animal studied	MNP evaluated	Tissue involved	Analytical method	Molecular mechanism	Pathophysiological and clinical effect
Li et al., [46]	Rats	Polystyrene (MP)	Myocardium, vascular wall	Histology, immunohistochemistry, molecular assays, western blot, transmission electron microscopy	Activation of Wnt/β‑catenin	Myocardial fibrosis and remodeling
Wei et al., [47]	Rats	Polystyrene (MP)	Myocardium	Histology, western blot, molecular assays, transmission electron microscopy	Activation of NLRP3/caspase‑1 (pyroptosis)	Inflammatory myocardial injury
Roshanzadeh et al., [57]	Rats	Polystyrene (NP, cationic)	Neonatal rat cardiomyocytes (in vitro)	In vitro imaging, mitochondrial potential assays, scanning electron microscopy, 3D optical diffraction tomography	Mitochondrial depolarization; Ca2 + alterations, oxidative stress	Loss of contractility, bradyarrhythmias
Wang et al., [59]	Mice	Polystyrene (NP neutral)	Vascular endothelium (mouse aorta, ex vivo/in vivo)	In vitro endothelial culture, immunostaining, fluorescence microscopy, RNA sequencing	Activation of JAK1/STAT3/Tissue Factor	Endothelial dysfunction, pro‑thrombotic state
Zhang et al., [30]	Mice	Polystyrene (NP, 100 nm)	Vascular wall	Histology, rRNA sequencing, GC-MS	Dyslipidemia; metabolic alterations, wall thickening	Atherosclerotic risk
Cary et al., [125]	Rats	Polyamide (MP)	Airways, lung, vascular wall	Bronchoalveolar lavage, cytokine assays, histology	Increased IL‑6, MCP‑1, increased CRP	Systemic inflammatory state (pro‑atherogenic)
Wen et al., [83]	Mice	Polystyrene (NP)	Vascular wall/plaque, liver	Histology, lipidomics, transmission electron microscopy	Altered hepatic lipid metabolism, inflammation	Accelerated atherosclerosis (ApoE−/−)
Zhong et al., [56]	Mice	Polystyrene (NP)	Vascular smooth muscle cells (plaque)	Cell culture immunofluorescence assays, histology, western blot, RNA sequencing	Upregulation of KIF15, vascular smooth muscle cells proliferation/migration	Plaque progression
Xiong et al., [54]	Mice	Polystyrene (NP)	Myocardium	Histology, molecular assays, eelectron microscopy, echocardiography	TNF‑α/NF‑κB and p38/MAPK; oxidative stress; apoptosis	Reduced LVEF, hypotension, eccentric remodeling

*ApoE* Apolipoprotein E, *CRP* C-reactive protein, *GC-MS* gas chromatography-mass spectrometry, *JAK1* Janus kinase 1, *KIF15* Kinesin family member 15, *LVEF* left ventricular ejection fraction, *MAPK* mitogen-activated protein kinase, *MCP-1* monocyte chemotactic protein 1, *MNP* microplastic and nanoplastics, *MP* microplastic, *NF- κB* nuclear factor kappa-light-chain-enhancer of activated B cells, *NLRP3* NLR family pyrin domain containing 3, *NP* nanoplastic, *RNA* ribonucleic acid, *STAT* signal transducer and activator of transcription, *TNF- α* tumor necrosis factor-α

**Table 2:** Principal human studies about MNPs detection in cardiovascular tissues with the associated molecular mechanism and the potential pathophysiological and clinical effect

Study: first author, year	MNP evaluated	Tissue involved	Analytical method	Molecular mechanism	Observed association or clinical correlation
Wu et al., [6]	Pigmented MP and fragments (various polymers)	Human arterial thrombi	Raman spectroscopy	Direct detection in thrombus, mechanism unknown	MNP detected in arterial thrombi (causality not established)
Rotchell et al., [50]	Polypropylene (fibers)	Great saphenous vein (surgical grafts)	µFTIR microscopy	Tissue detection, potential local irritation and inflammation	MNP detected in surgical vascular graft specimens (clinical significance unknown)
Yang et al., [51]	Various polymers (polystyrene, polyethylene, polycarbonate, polyethylene terephthalate, polyurethane, polyvinyl chloride, and polymethyl methacrylate)	Pericardium, epicardial fat, myocardium, left atrial appendage	LDIR spectroscopy	Tissue detection, mechanisms unknown	MNP detected in cardiac surgical tissues (no outcome correlation assessed)
Liu et al., [52]	Various polymers (polyethylene, polyethylene terephthalate, polyvinyl chloride, and polyamide-66)	Coronary, carotid, aortic tissues	Pyrolysis-gas chromatography mass spectrometry (Py-GC/MS)	Biomolecular detection; potential association with atherosclerosis	MNP presence in atherosclerotic tissues; associative relationship reported
Marfella et al., [5]	Various polymers (polyethylene, polyvinyl chloride)	Carotid plaques	µFTIR microscopy, Raman spectroscopy, Histology (electron microscopy)	Higher polymer burden associated with inflammation and reduced collagen	Patients with MNPs in plaque had an increased 3‑year risk of major adverse cardiac and cerebrovascular events (observational association)
Massie et al., [42]	Various polymers (polyethylene, polystyrene, polypropylene, acrylonitrile butadiene styrene, styrene-butadiene, polyvinylchloride, polyethylene terephthalate, poly(methyl methacrylate), polycarbonate, nylon 66, and nylon 6)	Common femoral artery plaques	Pyrolysis-gas chromatography mass spectrometry (Py-GC/MS)	Tissue detection, mechanisms unknown	Higher polymer concentrations observed in patients with chronic limb-threatening ischemia compared with claudication (cross-sectional association)

*MNP* microplastic and nanoplastics, *MP* microplastic, *NP* nanoplastic, *µFTIR* micro-fourier transform interferometer, *LDIR* laser direct infrared, *Py-GC/MS* pyrolysis-gas chromatography mass spectrometry

### Oxidative Stress and Endothelial Dysfunction

A frequently proposed mechanism linking MNPs to cardiovascular toxicity is the promotion of oxidative stress and endothelial dysfunction [62].

Experimental data from in vitro studies using cultured human umbilical vein endothelial cells (HUVECs) and murine endothelial cells exposed to PS-MNPs demonstrate particle internalization (particularly particles in the nano-size range and those with positively charged or hydrophobic surfaces) and oxidative stress responses [63].

In these in vitro systems, particularly with PS-NPs, internalization has been associated with mitochondrial dysfunction, ROS production, and antioxidant defense systems impairment leading to oxidative modification of lipids, proteins, and nucleic acids, compromising cellular homeostasis [64, 65].

Excessive ROS generation leads to reduced nitric oxide (NO) bioavailability, driven both by direct NO scavenging and oxidation of tetrahydrobiopterin (BH4), a cofactor essential for normal eNOS function. Loss of NO signaling impairs vasodilation and promotes vasoconstriction, leukocyte adhesion, and platelet activation [59].

MNPs-related structural effects include disruption of intercellular junctions, increased endothelial permeability, and alterations in cytoskeletal organization, changes that favour inflammation and vascular injury [59, 66]. Upregulation of adhesion molecules such as vascular cell adhesion protein 1 (VCAM-1) and intercellular adhesion molecule-1 (ICAM-1) facilitates monocyte recruitment and initiation of intimal inflammation, processes central to atherosclerotic plaque development [67]. Importantly, experimental studies in murine models, have demonstrated that exposure to PS MNPs directly induces endothelial ICAM-1 and VCAM-1 expression. and promoted leukocyte adhesion through NLRP3 inflammasome activation and NF-κB signaling. Similarly, in vitro exposure of endothelial cells to PS particles has been shown to enhance adhesion molecule expression and impair barrier integrity, supporting a direct pro-inflammatory endothelial effect of MNPs [59, 66]. Macrophages, monocytes, dendritic cells, and endothelial cells can internalize MNPs, leading to activation of pattern-recognition receptors, including Toll-like receptors (TLRs) and the NLRP3 inflammasome [68].

**Fig. 3:**
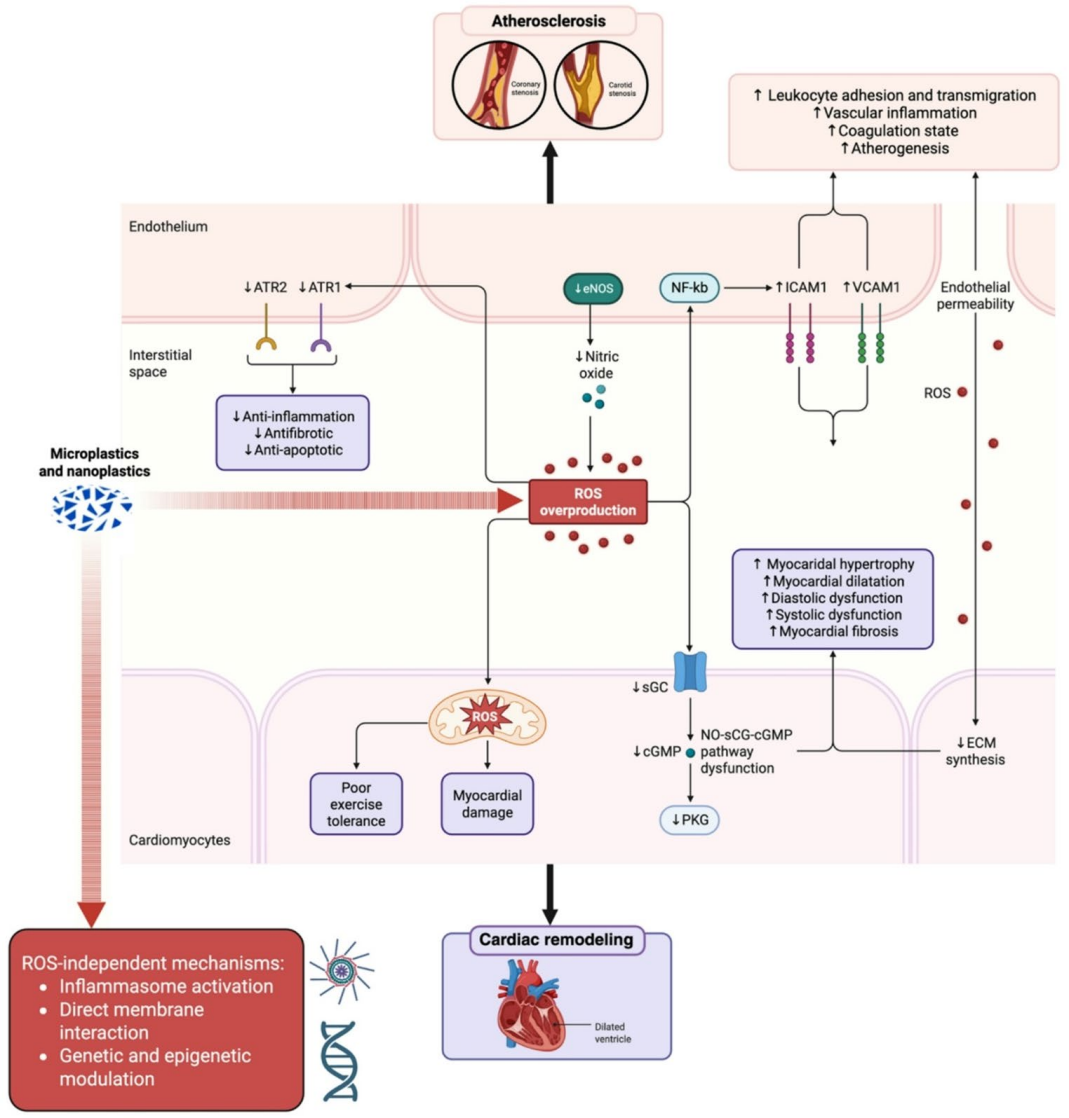
Proposed and experimentally derived mechanisms linking MNP exposure to cardiovascular effects. ROS overproduction represents one of the most described pathways, but ROS-independent mechanisms may also contribute to cardiovascular effects

Collectively, these findings suggest that MNP exposure may contribute to oxidative imbalance and endothelial perturbation, processes that are relevant to atherosclerotic initiation and progression (Fig. 3). Nevertheless, whether oxidative stress represents a primary driver or a secondary epiphenomenon in this context remains to be clarified.

### Systemic and Vascular Inflammation

Beyond oxidative stress, in vivo experimental studies have shown that MNPs exposure promotes inflammatory activation within both vascular and circulating immune compartments [69]. Importantly, several experimental studies have directly documented cytokine upregulation following MNP exposure. In rodent models, oral or inhalational exposure to PS-MNPs increased circulating and tissue levels of TNF-α, interleukin-1β, and interleukin-6, accompanied by vascular inflammatory infiltration and endothelial dysfunction leading to cardiovascular toxicity [47, 54, 59].

In murine in vivo exposure models, circulating immune cells isolated from MNP-exposed animals exhibit enhanced ROS generation, altered phagocytic capacity, and metabolic reprogramming toward glycolytic activation, a pattern characteristic of trained innate immunity. Furthermore, in murine models, chronic MNPs exposure has been shown to influence bone marrow haematopoiesis, skewing progenitor differentiation toward myeloid lineages, thereby sustaining chronic low-grade inflammation. Such changes may contribute to sustained low-grade systemic inflammation, even in the absence of ongoing exposure [4, 70].

In rodent models, a direct impact of PS-NPs on vascular stenosis was found, through alterations in lipid metabolism and thickening of the arterial wall [71]. These effects may be amplified in the presence of traditional cardiovascular risk factors such as dyslipidemia, hypertension, and metabolic dysfunction [72].

Taken together, these in vivo observations provide mechanistic evidence that MNPs can enhance inflammatory signaling within endothelial and immune cells, potentially intensifying vascular inflammation and contributing to atherogenesis, particularly in predisposed individuals, although human causal evidence remains limited.

### Genetic and Epigenetic Changes

Emerging evidence suggests that, beyond oxidative stress and inflammation, MNPs may induce genetic damage and epigenetic reprogramming, potentially contributing to long-term cardiovascular risk.

Experimental studies indicate that MNP exposure can promote deoxyribonucleic acid (DNA) strand breaks, chromosomal instability, and oxidative DNA lesions, largely mediated by excessive reactive oxygen species generation. Persistent oxidative stress may overwhelm DNA repair mechanisms, leading to genomic instability and activation of stress-responsive transcriptional programs [4, 73, 74]. Although direct human cardiovascular data remain limited, these mechanisms are well recognized contributors to vascular aging and atherogenesis.

In addition to direct genotoxic effects, MNPs appear capable of inducing epigenetic modifications [75]. In animal and cellular models, exposure to PS MPN has been associated with alterations in global and gene-specific DNA methylation patterns, particularly in pathways involved in inflammation, lipid metabolism, and cellular stress responses [76, 77]. Such epigenetic remodeling may result in sustained transcriptional activation of pro-inflammatory and pro-atherogenic genes, even after cessation of exposure.

Moreover, recent studies suggest that MNP exposure can modulate non-coding ribonucleic acid (RNA) expression, including microRNAs implicated in endothelial dysfunction and vascular remodeling. Dysregulation of microRNAs known to regulate NF-κB signaling, oxidative stress responses, and smooth muscle cell phenotype switching may provide a mechanistic bridge between environmental exposure, cardiotoxicity and accelerated atherosclerotic progression [78, 79].

Histone modifications have also been reported in experimental systems exposed to MPs, with changes in acetylation, methylation and lactylation, marks associated with immunotoxicity [4, 80]. These findings raise the possibility that MNPs may induce a form of environmentally driven “vascular memory,” analogous to metabolic memory described in diabetes, thereby amplifying cardiovascular susceptibility over time.

Although most current evidence derives from non-cardiac tissues or animal models, the convergence of genotoxic stress and epigenetic reprogramming with established cardiovascular pathogenic pathways strongly supports further investigation into MNP-induced genomic and epigenomic alterations as novel mechanisms of cardiovascular toxicity.

### Mitochondrial Dysfunction and Inflammatory Myocardial Injury

The myocardium is highly sensitive to disruptions in mitochondrial homeostasis, given its reliance on oxidative phosphorylation to sustain contractile function [81]. Experimental studies in neonatal rat cardiomyocytes exposed in vitro to PS-NPs indicate that MNPs can localize to cardiomyocytes and cardiac mitochondria following systemic exposure. Internalized particles are associated with increased mitochondrial ROS production, impaired electron transport chain activity, and reduced ATP generation [57].

These mitochondrial alterations are accompanied by depolarization of mitochondrial membrane potential, release of pro-apoptotic factors, and activation of intrinsic cell death pathways. Transmission electron microscopy studies reveal that cardiomyocytes exposed to MNPs exhibit swollen mitochondria, and signs of mitophagy failure [2].

Functional consequences include reduced left ventricular contractility, impaired diastolic relaxation, and altered calcium handling, consistent with subclinical myocardial dysfunction. In murine models exposed to PS-MPs, most commonly via oral administration in drinking water or controlled inhalational exposure, prolonged exposure has been associated with inflammatory cell infiltration within myocardial tissue and increased expression of pro-inflammatory mediators [47].

Although direct clinical evidence in humans remains limited, the combination of mitochondrial disruption, cellular injury, and remodeling observed in preclinical models suggests that MNPs may contribute to myocardial vulnerability and functional decline, particularly in the context of underlying cardiovascular disease, where mitochondrial dysfunction and maladaptive remodeling are central determinants of heart failure (HF) progression [81].

**Fig. 4:**
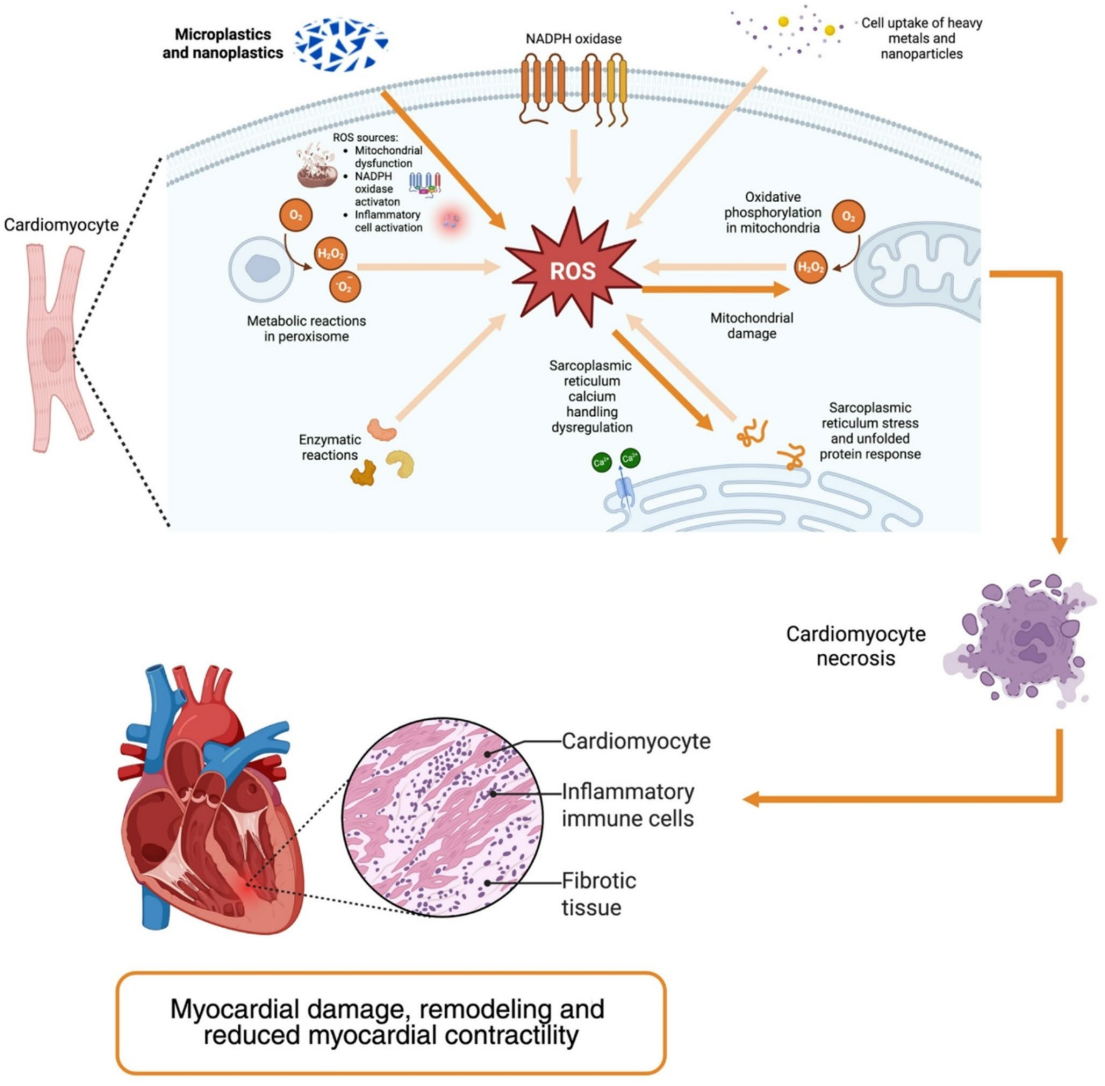
Microplastics and nanoplastics molecular mechanisms leading to cardiomyocyte homeostasis dysregulation and impaired myocardial function

In summary, the impairment of mitochondrial integrity and function by MNPs may initiate a cascade of events culminating in reduced myocardial contractility, arrhythmic vulnerability, and remodeling changes (Fig. 4) that are mechanistically consistent with pathways implicated in HF, while acknowledging that direct evidence of HF phenotypes in humans remains unavailable.

## Is There Evidence of Involvement in Disease? Microplastics and Nanoplastics in Different Pathophysiological Scenarios

### Atherosclerosis Development and Plaque Progression

Experimental and early translational evidence suggests that MNPs may influence the development and progression of atherosclerotic cardiovascular disease. However, current data are largely derived from controlled experimental systems employing specific polymers and exposure doses that may not fully reflect real-world human exposure. Thus, while mechanistic plausibility is strong, direct evidence of causality in human atherosclerosis remains limited.

The pathways described above, oxidative stress, endothelial dysfunction, innate immune activation, and mitochondrial injury, intersect directly with those known to drive atherosclerotic plaque initiation and evolution [72]. Endothelial dysfunction and increased leukocyte adhesion associated with MNP exposure may promote monocyte recruitment into the intima and the formation of foam cells.

Over time, persistent low-grade inflammation may support smooth muscle cell phenotypic switching, extracellular matrix remodeling, and plaque growth. These processes are consistent with early and stable stages of coronary atherosclerosis [82].

In ApoE⁻/⁻ mice receiving oral PS-NPs (~ 100 nm) in drinking water for several weeks, plaque areas showed reduced collagen content, increased macrophage infiltration, and upregulation of matrix metalloproteinases compared with controls, features that characterize vulnerable plaque phenotype [83, 84].

Increased oxidative stress may further contribute to necrotic core expansion and fibrous cap thinning. These structural changes align with higher propensity for plaque disruption (Fig. 3). Taken together, these findings support the hypothesis that NPs can interact with vascular, smooth muscle, and immune cells inducing molecular and cellular changes associated with plaque progression and instability. However, the extent to which these experimental observations translate into clinically meaningful effects in humans is still uncertain. Indeed, it is important to emphasize that atherosclerosis is a multifactorial disease involving metabolic, hemodynamic, genetic, and environmental determinants [85]. MNP exposure, if contributory, is likely to represent one component within a broader network of interacting risk factors rather than a single dominant mechanistic driver. Current evidence supports biological plausibility, but substantial gaps remain regarding dose-response relationships, chronic low-dose exposure effects, and inter-individual susceptibility.

### Atherothrombosis

Inflammatory activation and platelet-endothelium interactions linked to MNP exposure may contribute to enhanced platelet reactivity and pro-thrombotic signaling. In murine models exposed intravenously to aminated PS-NPs, increased fibrin deposition, accelerated thrombus formation, and elevated circulating markers of coagulation pathway activation have been documented [49, 66, 86]. In a human study, the mass concentrations, polymer types, and physical properties of MPs in human thrombi surgically retrieved from both arterial and venous systems were investigated. Among the 15 identified MPs, PE was the predominant particle found. No significant association between the concentration of MPs and the size of thrombi was found and the concentration of MPs was similar between arterial and venous system. Notably, higher concentrations of MPs were associated with higher ischemic stroke severity. However, the mean diameter of MPs found was 35.6 μm and, considering that particles in the 30–40 μm range exceed the size typically considered capable of systemic translocation, the possibility of contamination or passive entrapment rather than active biological penetration should be strongly considered [40].

To summarize, early human studies reporting the presence of MNPs in atherosclerotic plaques and coronary thrombi provide histological evidence of MNP localization within vascular tissues [5, 40], but do not yet establish causality between MNP accumulation and clinical events. Further research is required to determine whether MNP burden correlates with plaque vulnerability, ischemic events, or treatment response.

Overall, current evidence suggests that MNPs may contribute to both chronic atherosclerotic disease progression and acute plaque destabilization-related events, particularly in individuals with underlying cardiovascular risk factors or systemic inflammation.

### Chronic Cardiac Remodeling and Functional Impairment

Beyond early inflammatory injury and mitochondrial dysfunction, prolonged MNP exposure in experimental models has been associated with structural myocardial remodeling characterized by interstitial fibrosis and progressive contractile impairment [72]. Preclinical models demonstrate that MNP exposure can modulate cardiomyocyte metabolism, impair mitochondrial energetics, and promote cellular stress responses associated with progressive myocardial dysfunction [87].

Mitochondrial injury induced by MNP internalization may reduce ATP availability and disrupt calcium handling, contributing to impaired contractile reserve and diastolic relaxation. These effects are accompanied by activation of pro-fibrotic pathways, including TGF-β signaling and extracellular matrix deposition [46]. Resulting interstitial fibrosis and capillary rarefaction are consistent with the structural substrate seen in early HF phenotypes, particularly heart failure with preserved ejection fraction (HFpEF). In human iPSC-derived cardiomyocytes (hiPSC-CMs), prolonged exposure to low concentrations of PS particles results in significant alterations of contractile properties, impaired calcium cycling, and increased expression of pro-BNP [88].

Metabolic stress responses also appear to involve shifts from oxidative phosphorylation to glycolytic metabolism, which mirror patterns observed in pressure overload, metabolic syndrome, and air pollution–associated cardiac injury [89–91]. In murine models exposed to PS-MNPs, alterations in cardiac metabolism and autonomic balance have been observed, including increases in sympathetic tone and changes in heart rate variability, which may further influence ventricular relaxation, myocardial oxygen demand, and arrhythmogenic potential [92]. While direct clinical evidence remains limited, the convergence of mitochondrial stress, metabolic reprogramming, and fibrotic remodeling in experimental studies supports the biological plausibility that MNP exposure could contribute to the development or progression of HF, although longitudinal human data are currently lacking.

## Therapeutic and Preventive Implications

The most immediate strategy to mitigate the harmful impact of MNPs involves a non-pharmacologic preventive strategy with public health environmental and behavioural interventions aimed at reducing plastic production and, therefore, individual exposure, particularly among potentially vulnerable groups such as individuals with pre-existing cardiovascular or metabolic disease, older adults, pregnant women, children, and occupationally exposed populations. These populations may exhibit heightened susceptibility due to impaired barrier function, comorbid inflammation, or cumulative lifetime exposure.

Given that MNPs primarily enter the body through ingestion and may translocate across the gut barrier, interventions aimed at minimizing exposure to plastic-packaged food and beverages represent the first potential approach. Furthermore, preserving gastrointestinal integrity could limit systemic dissemination [29]. Preclinical data suggest that gut barrier dysfunction plays a key role in the systemic inflammatory response triggered by MNPs [30]. Thus, strategies involving prebiotics, probiotics, or dietary fibres, capable of enhancing mucosal defense and modulating microbiota composition, might serve as adjunctive tools to reduce cardiovascular risk linked to plastic exposure [93]. Moreover, the implementation of washing machine filtration systems designed to capture synthetic textile fibers before wastewater discharge could be a further effective strategy [94].

From a therapeutic standpoint, given the current limitations in exposure quantification, causal inference, and translational extrapolation, it would be premature to propose modification of established cardiovascular therapeutic strategies based on MNP exposure. Existing guideline-directed medical therapy has demonstrated benefit in populations already ubiquitously exposed to environmental MNPs. Therefore, the most appropriate clinical response at this stage lies in exposure mitigation and further research rather than pharmacologic intensification. However, while current cardiovascular therapies were not developed with environmental toxicant exposure in mind, growing evidence that MNPs accumulate in vascular and myocardial tissues raises an important translational question: could environmental plastic burden modify therapeutic efficacy or contribute to residual cardiovascular risk despite optimal guideline-directed therapy? Rather than reiterating standard treatment algorithms, this section explores how MNP-driven oxidative stress, inflammation, endothelial dysfunction, platelet activation, and metabolic reprogramming may interact with existing pharmacological strategies. Understanding these interactions may open new perspectives in precision cardiovascular medicine, where environmental exposure becomes an additional dimension of risk stratification and therapeutic modulation.

### Atherosclerosis Development and Progression

Beyond environmental exposure reduction, optimal management of classical cardiovascular risk factors (including obesity, diabetes mellitus, dyslipidemia, arterial hypertension and smoking) remains essential. These conditions may interact with MNP exposure through shared inflammatory and oxidative mechanisms relevant to atherosclerosis progression [95].

Hence, optimal medical therapy should target not only metabolic disorders but also the modulation of vascular inflammation. High-intensity statins remain the cornerstone, not only due to their LDL-C lowering capacity but also for their well-documented anti-inflammatory effects [96]. Moreover, PCSK9 inhibitors further augment this protective effect in patient with high risk cardiovascular profile [97]. Notably, experimental data suggest that NPs interfere with hepatic lipid metabolism and cholesterol efflux pathways [60]. However, there is currently no clinical evidence indicating that MNP exposure attenuates the benefits of statins or other lipid-lowering therapies.

Also, considering that MNP exposure chronically activates NF-κB signaling and inflammasome pathways, residual vascular inflammation may persist despite adequate LDL-C reduction [69, 98]. Whether MNP exposure modifies therapeutic response remains unknown. At present, standard risk-based management remains the appropriate clinical approach.

Several cardiovascular therapies, including statins, glucagon-like petide-1 (GLP-1) receptor agonists, renin-angiotensin-aldosterone-system inhibitors, and selected anti-inflammatory agents, exert pleiotropic anti-inflammatory effects that may theoretically intersect with pathways activated by MNP exposure. However, no evidence currently supports therapy modification based on environmental exposure. Future directions must include the development of non-invasive biomarkers to detect MP burden in humans and identify individuals at high risk of accelerated atherosclerosis. Moreover, it remains to be elucidated whether the vascular effects attributed to MNPs exposure are independent or confounded by coexisting risk factors.

### Atherothrombosis

The recognition that MNPs may contribute to the onset and progression of atherothrombosis carries significant implications for both acute and long-term management. Percutaneous coronary intervention (PCI), the cornerstone of coronary atherothrombosis treatment, is particularly sensitive to alterations in thrombogenicity, vascular healing, and response to antithrombotic agents. Although experimental studies suggest that certain MNPs can modulate inflammatory and haemostatic pathways [99, 100], there is currently no clinical evidence demonstrating that environmental MNP exposure alters PCI outcomes.

From a research perspective, PCI-derived thrombus samples may offer an investigational opportunity to explore possible associations between MNP presence and thrombus composition [40]. In the post-PCI setting, chronic exposure to MNPs could perpetuate platelet activation and low-grade vascular inflammation, thereby promoting stent thrombosis, neo-atherosclerosis and in-stent restenosis. Whether chronic MNP exposure influences stent healing or thrombotic risk remains speculative and unproven.

Another underexplored domain is the interaction between MNPs and drug-eluting stent (DES) technology. The therapeutic efficacy of DES depends on precise tightly regulated release of antiproliferative agents, which are governed by polymer composition and degradation dynamics. Experimental evidence from nanotoxicology studies indicates that NPs can adsorb small hydrophobic drug molecules onto their surface and alter local oxidative and inflammatory microenvironments [23]. In addition, oxidative stress is known to influence polymer degradation and drug-release kinetics in polymer-based delivery systems [101, 102]. Therefore, by adsorbing drug molecules or modulating local redox conditions, NPs could theoretically interfere with DES polymer integrity and drug elution profiles, potentially modifying vascular healing dynamics. While direct evidence in coronary stent models is currently lacking, this hypothesis is biologically plausible and warrants dedicated translational investigation.

From a pharmacological standpoint, MNP-driven platelet hyperreactivity raises questions about the optimal antithrombotic strategies. Ex vivo data show that PS- NPs enhance platelet aggregation even in the presence of aspirin, suggesting that conventional cyclooxygenase inhibition may be inadequate [69]. Whether P2Y_12_ receptor inhibition is similarly affected remains unknown, and the clinical relevance of MNPs-related potential platelet hyper-reactivity remains uncertain. However, if NPs enhance platelet reactivity [103], conventional antiplatelet strategies may not fully neutralize MNP-driven thrombogenicity. This raises the possibility that environmental exposure could influence optimal intensity or duration of antithrombotic therapy, particularly in high-risk individuals.

Finally, long-term management (including anti-inflammatory and lipid management strategies) may need to integrate an environmental dimensions. Although avoidance of MNPs is difficult in modern societies, public health interventions aimed at reducing plastic pollution may confer indirect cardiovascular benefits. Clinically, recognizing environmental exposure as a potential risk modifier could refine stratification algorithms and guide the intensity of secondary prevention measures [104].

In summary, the intersection between MNP exposure and atherothrombosis management spans multiple domains: stent healing, antithrombotic therapy, anti-inflammatory strategies, lipid management, and risk stratification. Although current evidence remains preliminary, future clinical studies will be crucial to determine whether environmental MNP exposure should be explicitly considered in therapeutic decision-making.

### Myocardial Damage and Remodeling

Although current management of cardiac dysfunction and remodeling do not yet address environmental pollutants such as MNPs, preclinical studies have reported myocardial effects associated with certain MNP exposures.

Several antioxidant compounds have been shown in experimental models to mitigate oxidative and inflammatory responses induced by MNPs [105, 106]. Similarly, resveratrol has been reported to exert cardioprotective effects by modulating oxidative stress pathways and downregulating inflammatory mediators [107, 108]. These compounds act at the crossroads of redox imbalance and inflammatory signaling, two pivotal drivers of plastic-related myocardial injury.

Some pharmacological agents already employed in HF management may counteract the harmful effects of MNPs. Sodium-glucose cotransporter 2 inhibitors (SGLT2i), angiotensin receptor–neprilysin inhibitors (ARNI), and mineralocorticoid receptor antagonists (MRA) have all demonstrated anti-inflammatory and antioxidant effects beyond their hemodynamic actions [109–112]. While direct evidence linking ARNI or MRA to mitigation MNP toxicity is currently lacking, recent preclinical studies suggest that SGLT2i may offer protection against plastic-induced cardiac injury through attenuation of oxidative stress and inflammation, positioning them as potential pharmacological allies in this emerging field of environmental cardiology [113].

Given that MNP-induced myocardial injury appears to involve mitochondrial dysfunction and oxidative stress, therapies with pleiotropic antioxidant and anti-inflammatory properties (such as SGLT2i) may exert differential protective effects in exposed individuals [114]. Whether environmental toxicant burden contributes to variability in heart failure progression or therapeutic response represents a largely unexplored research frontier.

In this framework, MNP exposure may represent a previously unrecognized contributor to residual cardiovascular risk, potentially influencing pharmacodynamic responses and long-term outcomes. Integrating environmental toxicology into therapeutic decision-making underscores the need to consider environmental burden as a potential modifier of cardiovascular therapy effectiveness. As global plastic production and environmental dispersion continue to rise, longitudinal studies will be necessary to determine whether escalating exposure modifies long-term treatment effectiveness or identifies subgroups with differential therapeutic response.

## Knowledge Gaps and Future Research Directions

Although evidence now demonstrates that MNPs are detectable in human cardiovascular tissues, the magnitude and clinical significance of their contribution to cardiovascular disease remains unclear. Addressing this knowledge gap will require coordinated experimental, epidemiological, and clinical investigation.

### Limitations of Polystyrene-Based Experimental Models

Most experimental studies rely on commercially available PS nanospheres, which are monodisperse, spherical, and non-weathered. Although these models have been instrumental for mechanistic discovery, they poorly reflect the physicochemical diversity of environmentally derived plastics, which are heterogeneous in size, shape, polymer composition, and surface chemistry [115]. Furthermore, PS represents only a fraction of environmental plastic burden, whereas PE, PP, and PET are more prevalent in environmental and human biomonitoring studies [116]. The biological responses to different polymers, surface chemistries, and aging processes may vary significantly, limiting the generalizability of PS-based findings. Future work should incorporate aged particles, mixed polymers, and environmentally realistic exposure paradigms to enhance translational relevance.

### Methodological and Interpretative Challenges in MNP-Cardiovascular Research

Several critical uncertainties must be acknowledged to balance the interpretation of emerging data linking MNP exposure to cardiovascular disease.

First, analytical detection methods remain imperfect. Pyrolysis-gas chromatography-mass spectrometry (Py-GC/MS), while widely used, may overestimate polymer burden through detection of shared hydrocarbon or fatty acid derivatives, potentially inflating MNP quantification [117]. Similarly, identification of suspect particles in biological tissues is subject to spectral ambiguity and contamination risk, particularly for particles in the > 200–500 nm range, whose systemic translocation from pulmonary or gastrointestinal barriers remains biologically debated [118].

Second, human association studies are inherently vulnerable to confounding. Individuals with higher inhalational MNP exposure are also likely to experience greater exposure to co-pollutants such as particulate matter (PM2.5), nitrogen oxides, and volatile organic compounds. Similarly, dietary sources enriched in MPs, such as ultra-processed foods, may independently confer cardiometabolic risk unrelated to MNP content. Disentangling particle-specific effects from correlated environmental and lifestyle factors remains a major epidemiological challenge [119].

Third, the relative contribution of particle-induced biological responses versus effects mediated by plastic additives (such as plasticizers, stabilizers) or adsorbed environmental contaminants has not been fully delineated [120]. This distinction has substantial regulatory implications.

Finally, current tissue-based studies are unable to determine the environmental source of detected MNPs, limiting source attribution and targeted preventive strategies. Improved environmental monitoring, standardized detection methodologies, and development of safer material alternatives will be essential components of future intervention frameworks.

Taken together, while systemic access of MNPs to vascular and cardiac tissues appears biologically plausible, the magnitude of their contribution to disease progression remains incompletely defined and requires cautious interpretation.

### Need for Standardized Reporting and Methodological Harmonization

A major obstacle to reproducibility and cross-study comparability in the MNP field is the lack of standardized reporting of experimental parameters. Variability in polymer characterization, particle size distribution, surface chemistry, dose metrics, exposure route, and analytical detection methods significantly complicates interpretation and translational extrapolation. Recent recommendations have emphasized the need for harmonized reporting frameworks and minimum information standards for MPs research to improve reproducibility and data integration across laboratories [121]. Adoption of such guidelines will be essential to strengthen causal inference and facilitate meaningful comparison between experimental systems and human exposure studies.

### Need for Longitudinal Studies and Clinical Trials

Despite growing preclinical evidence linking MNPs to cardiovascular toxicity, clinical data remain limited [19]. Currently, available insights derive from experimental models that elucidate molecular mechanisms but cannot establish causal relationships in humans. Current clinical and observational studies, are constrained by major limitations, including imprecise quantification of real-world exposure, multiple environmental confounders, and the absence of long-term follow-up [122]. To fill the gap in this field, robust longitudinal cohort studies are urgently needed to monitor MNPs exposure over time and evaluate its relationship with major adverse cardiovascular events. These investigations should integrate comprehensive environmental exposure assessments, through analysis of biological matrices (such as blood, urine, or cardiovascular tissues), and explore dose-response interactions with traditional risk factors (such as smoking, diabetes, obesity, and hypertension) [18].

However, significant methodological challenges persist, including the lack of validated biomarkers of internal exposure, limited standardization of analytical techniques, and geographic and cultural variability in exposure, all of which complicate study harmonization. Given the complexity of designing interventional studies to reduce plastic exposure, fostering multidisciplinary research networks involving cardiology, toxicology, and environmental science is essential [123].

Only through such coordinated efforts can theoretical concerns evolve into evidence-based strategies informing future clinical and public health policies.

### Development of Biomarkers of Exposure and Cardiovascular Injury

Although the cardiovascular consequences of MNP exposure may manifest only after prolonged accumulation, global plastic production and environmental dispersion are projected to continue rising under business-as-usual scenarios, with annual plastic output expected to increase substantially in the coming decades [124]. Therefore, identifying early biomarkers capable of detecting MNP-related cardiovascular injury represents a crucial preventive goal.

In vivo animal studies have shown that exposure to PS MNPs can disrupt lipid metabolism.

In murine models, high-dose exposure to 100 nm PS nanoparticles (100 µg/mL in drinking water) resulted in vascular wall thickening, increased serum low-density lipoprotein, triglycerides, total cholesterol, alanine aminotransferase, and aspartate aminotransferase, together with reduced high-density lipoprotein levels [78].

In a Wistar rat model, oral exposure to PS-MPs (administered via drinking water in three concentrations of 0.5, 5 and 50 mg/L) was associated with cardiac damage (showed by increased levels of creatine-kinase MB and cardiac troponin I), oxidative stress (as indicated by increased levels of malondialdehyde and decreased activity of superoxide dismutase, glutathione peroxidase and catalase) and cardiomyocytes pyroptosis mediated by activation and increased expression of interleukin-1β and interleukin-18 [47].

Similarly, inhalational exposure to polyamide MPs in rat models induced systemic inflammation, with elevated interleukin-6 and trends toward increased C-reactive protein (CRP) [125]. While such findings suggest that inflammatory biomarkers may reflect systemic responses to MNP exposure, markers such as CRP and interleukin-6 are inherently non-specific and influenced by numerous metabolic, infectious, and environmental factors [126, 127]. Routine screening based on these parameters alone would therefore lack specificity and may not be clinically practical or cost-effective without more precise exposure assessment tools.

To summarize, traditional cardiovascular biomarkers such as lipid profile and inflammatory markers remain essential tools for global cardiovascular risk assessment. However, they do not provide specific information regarding MNP exposure. At present, no validated clinical biomarkers exist to quantify individual MNP burden or to directly attribute cardiovascular alterations to environmental plastic exposure. The development of exposure-specific and mechanistically informative biomarkers represents an important unmet need in this field.

### Clinical Scenarios Awaiting Evidence

Despite the expanding evidence linking MNPs to cardiovascular risk, several cardiovascular clinical scenarios remain largely unexplored. One striking gap concerns valvular heart disease (VHD). Fibrocalcific aortic stenosis and degenerative mitral valve disease are highly prevalent in aging societies [128], yet no study to date has systematically investigated the potential presence or role of MNPs within valvular tissue or their contribution to processes such as fibro-calcific remodeling. Given that valvular degeneration involves oxidative stress, chronic inflammation, and osteogenic transformation of interstitial cells, pathways similarly activated by MNPs in vascular and myocardial models, it is biologically plausible that chronic exposure could accelerate fibro-calcific remodeling [129]. By analogy, it is biologically plausible that chronic exposure to these particles could accelerate or exacerbate valvular calcification, but this remains a hypothesis awaiting direct validation [18].

Comparable uncertainties exist for other widespread conditions such as hypertension and diabetes mellitus, both major contributors to cardiovascular morbidity. The capacity of MNPs to impair endothelial nitric oxide availability, increase vascular stiffness, and induce oxidative stress or mitochondrial dysfunction suggests potential pathogenic intersections with these disorders [130]. Yet, despite compelling mechanistic plausibility, clinical data remain virtually absent, leaving the question unresolved, as both processes might theoretically be influenced by MNP exposure. Ultimately, the shared mechanisms of oxidative stress, endothelial activation, mitochondrial disruption, and chronic inflammation observed in MNP-exposed models represent unifying biological themes across multiple cardiovascular and metabolic diseases [131]. While clinical data remain limited, the mechanistic rationale for concern is compelling and underscores the urgent need to address these neglected scenarios in future translational and epidemiological research [132].

### Conclusions - The Missing Link: Does Removal of the Noxa Removes the Disease?

Given the complexity of cardiovascular diseases, one of Koch´s criteria, that the disease is only present in the presence of the noxa, can definitely not be proven. Nonetheless, MNPs are now recognized as widespread, persistent environmental contaminants with demonstrated bioavailability in human tissues. Experimental studies demonstrate that they cross biological barriers, accumulate in vascular and myocardial tissues, and trigger oxidative stress, endothelial dysfunction, mitochondrial injury, and systemic inflammation. Through these mechanisms overlap with fundamental pathways of atherosclerotic development and progression, atherothrombosis, and myocardial dysfunction, suggesting that MNPs may act as emerging cardiovascular risk factor.

Across clinical settings, their impact appears multifaceted and can be associated to three main clinical scenarios. Through vascular inflammation, impaired cholesterol handling, and macrophage polarization MNPs may accelerate atherosclerotic progression leading to chronic coronary syndrome onset. As explored, MNPs may promote plaque rupture and erosion, enhance platelet activation, and accelerate thrombus formation and, for this reason, we might argue a potential role in the acute coronary syndromes’ pathogenesis. Finally, MNPs have been linked to impaired mitochondrial function, contractile dysfunction, and maladaptive remodeling, potentially contributing to HF development.

While mechanistic evidence is increasingly robust, clinical validation remains limited. The detection of MNPs in human plaques and cardiovascular tissues provides direct evidence of vascular tissue presence, underscoring the need for further investigation into clinical significance.

Moving forward, standardized exposure metrics, validated biomarkers of internal burden, and prospective clinical studies will be essential to determine whether MNP accumulation represents a manageable cardiovascular risk factor. Integrating MNP exposure into preventive cardiology frameworks will require close collaboration across cardiology, toxicology, environmental health, and regulatory science.

In summary, MNPs represent an emerging cardiovascular threat and an insidious cardiovascular risk factor, bridging the domains of environmental science and cardiovascular medicine. Understanding how these ubiquitous particles interact with vascular, myocardial, and systemic pathways will require rigorous, multidisciplinary investigation.

As the global burden of cardiovascular disease continues to rise, recognizing and investigating environmental contributors such as MNPs may open new pathways for prevention, risk stratification, and targeted intervention. Certainly, integrating environmental exposure into the cardiovascular risk paradigm may not only refine prevention and therapy, but also expand the boundaries of what constitutes cardiovascular health in the twenty-first century.

## Data Availability

No datasets were generated or analysed during the current study.
